# Supplementary material for: Differential Effects of Dietary versus Exercise Intervention on Intrahepatic MAIT Cells and Histological Features of NAFLD
Source: Nutrients. 2022 May 25;14(11):2198. doi: 10.3390/nu14112198 (PMC9182470; doi:10.3390/nu14112198)
Supplement: Supplementary file 1 [file nutrients-14-02198-s001.zip › nutrients-1698839-supplementary.pdf]

SUPPLEMENTARY FIGURES AND TABLES

Differential effects of dietary versus exercise intervention on intrahepatic MAIT cells and histological features of NAFLD

Naimimohasses S, O'Gorman P, Wright C, Ni Fhloinn D, Holden D, Conlon N, Monaghan A, Kennedy M, Gormley J, Beddy P, Finn SP, Lysaght J, O'Sullivan J, Dunne MR, Norris S* and Moore JB*

*Corresponding authors: Suzanne Norris snorris@tcd.ie and J. Bernadette Moore J.B.Moore@leeds.ac.uk

Table of Contents

Figure S1. Individual liver enzyme changes 2

Figure S2. Energy intakes and dietary quality 3

Table S1: Energy and macronutrient intakes 4

Figure S3. Individual histological changes 5

Figure S4. CD69 and CD95 expression in MAIT cells 6

FIGURE S1

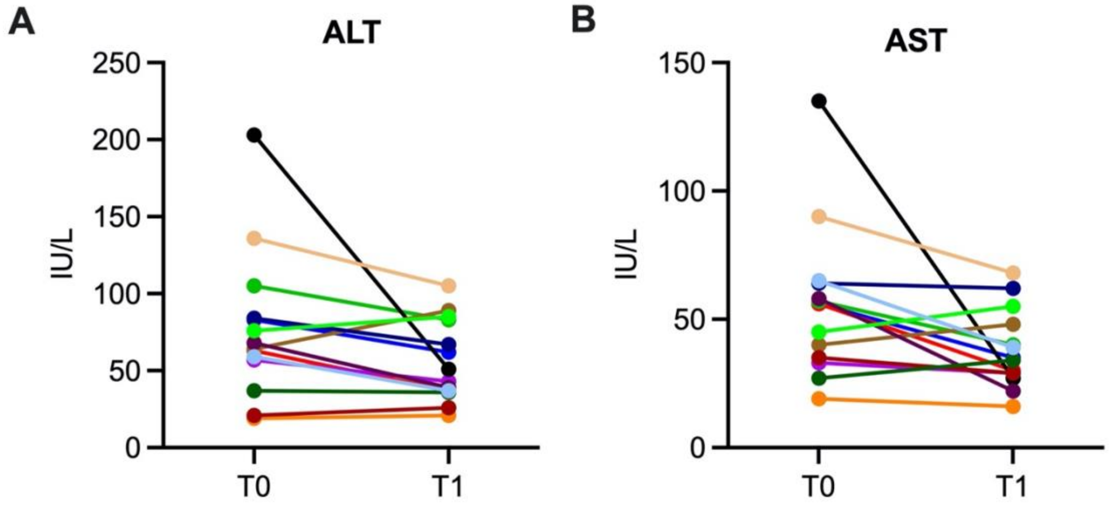

Figure S1. Individual liver enzyme changes in control group participants. (A) ALT and (B) AST levels at baseline assessment (T0) and after 12 weeks of standard of care (T1).

FIGURE S2

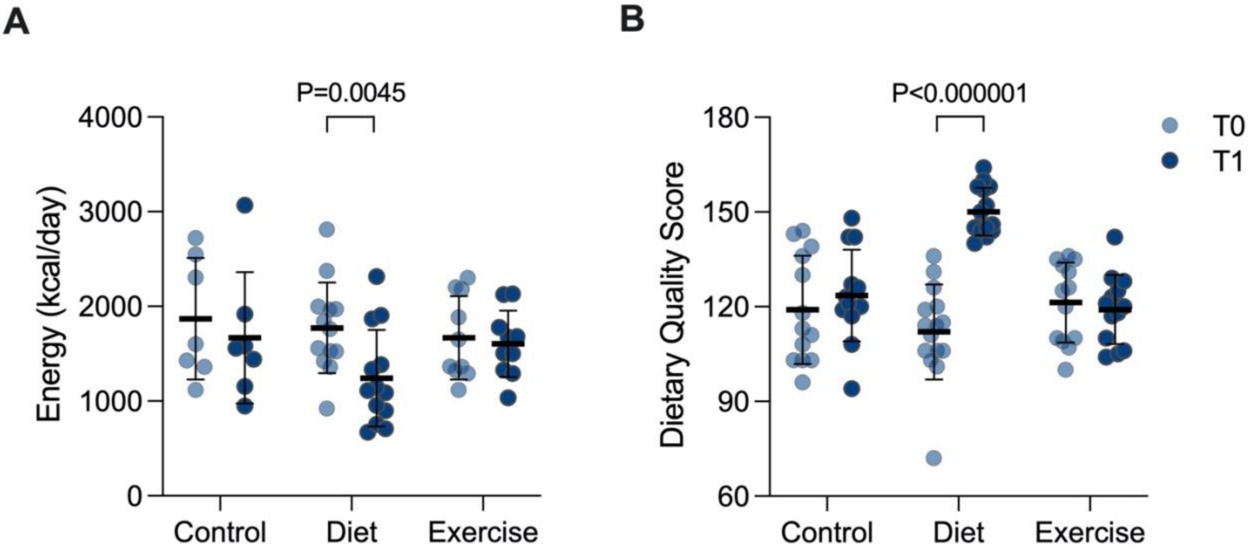

Figure S2. Energy intakes and dietary quality before and after diet or exercise intervention. (A) Average daily energy intakes calculated from detailed four-day diet diaries from control (n=7), nutrition (n=13) and exercise (n=10) group participants who completed diaries both before (T0) and after (T1) 12 weeks intervention. (B) Dietary quality assessed through food frequency questionnaire interviews given to control (n=13), diet (n=15) and exercise (n=13) group participants before and after intervention. Data were assessed for normality and analysed by multiple paired t tests. Solid black line and whiskers show mean and standard deviation.

TABLE S1

Table S1: Energy and macronutrient intakes calculated from paired 4-day diet diaries.

	Control (n=7)			Diet (n=13)			Exercise (n=10)		
**Intakes[1]**	**T0**	**T1**	**P value**	**T0**	**T1**	**P value**	**T0**	**T1**	**P value**
**Energy (kcal/d)**	1869.6 ± 640.8	1667.9 ± 692.2	0.4342	1772.5 ± 479.1	1241.7± 509.3	**0.0045**	1668.0 ± 440.3	1605.0 ± 350.9	0.5907
**Protein (g/d)**	88.3 ± 17.8	77.7 ± 23.6	0.1563	71.8 ± 16.5	73.7 ± 21.6	0.7501	77.4 ± 24.0	72.7 ± 18.2	0.3579
**Protein (%TE)**	19.9 ± 4.5	19.3 ± 3.3	0.5504	16.6 ± 2.5	24.9 ± 5.4	**0.0003**	18.6 ± 3.5	19.3 ± 3.4	0.8272
**Fat (g/d)**	73.3 ± 36.0	65.7 ± 43.3	0.6250	71.1 ± 28.9	54.0 ± 27.9	0.0803	64.1 ± 26.7	66.1 ± 19.1	0.7648
**Fat (%TE)**	33.8 ± 8.3	33.2 ± 10.0	0.8826	35.6 ± 8.0	38.4 ± 9.6	0.3241	33.6 ± 7.6	36.9 ± 6.5	0.2382
**SFA (g/day)**	26.8 ± 15.9	21.6 ± 15.8	0.3925	24.9 ± 11.9	16.5 ± 6.8	**0.0260**	24.4 ± 13.3	24.3 ± 9.4	0.9636
**SFA (%TE)**	11.9 ± 4.1	10.6 ± 3.6	0.3882	12.5 ± 4.3	12.4 ± 4.4	0.9619	12.5 ± 4.1	13.4 ± 3.2	0.4474
**Carbohydrate (g/d)**	213.0 ± 80.0	192.7 ± 77.1	0.5056	184.1 ± 40.1	108.9 ± 48.4	**0.0001**	197.2 ± 54.6	180.2 ± 45.9	0.4045
**Carbohydrate (%TE)**	46.3 ± 9.9	47.2 ± 12.0	0.8082	42.9 ± 9.2	35.5 ± 9.6	**0.0475**	47.9 ± 7.4	44.9 ± 5.0	0.3037
**Total sugars (g/d)**	83.0 ± 42.5	61.7 ± 39.5	0.2448	74.8 ± 30.5	41.8 ± 23.9	**0.0024**	80.8 ± 44.0	69.8 ± 32.7	0.3343
**Total sugars (%TE)**	17.5 ± 5.4	14.8 ± 6.5	0.3020	17.1 ± 5.6	13.4 ± 5.3	0.0847	18.7 ± 6.4	17.0 ± 5.4	0.3393
**Dietary fibre[2] (g/d)**	5.2 ± 4.0	7.3 ± 2.8	0.1643	8.7 ± 5.2	7.9 ± 7.3	0.4533	8.0 ± 3.3	7.5 ± 3.4	0.5899
**Sodium (g/d)**	3.1 ± 1.2	2.5 ± 1.4	0.2099	2.4 ± 0.6	2.3 ± 0.8	0.6770	2.6 ± 1.0	2.5 ± 0.88	0.6441

[1]Data were tested for normality using Shapiro-Wilk test and presented as mean ± standard deviation with differences assessed by paired t test. [2]AOAC method. Abbreviations: AOAC, American Association of Analytical Chemists; SFA, saturated fatty acids; TE, total energy.

FIGURE S3

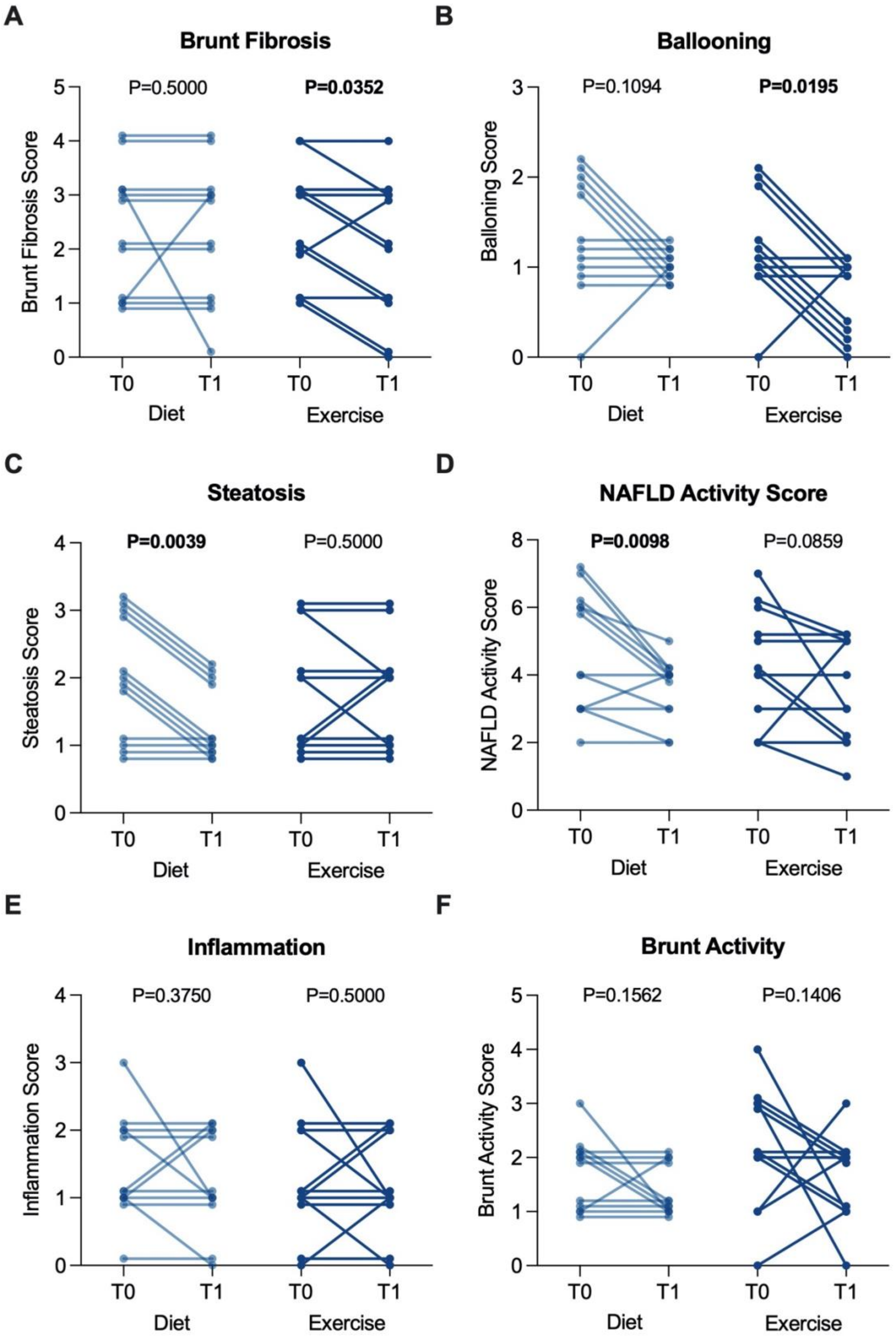

Figure S3. Individual histological changes in response to 12 weeks diet or exercise intervention. (A) Fibrosis, (B) Ballooning, (C) Steatosis, (D) NAFLD Activity Score, (E) Lobular inflammation, and (F) Brunt Activity Score. T0, baseline assessment; T1, post intervention assessment.

FIGURE S4

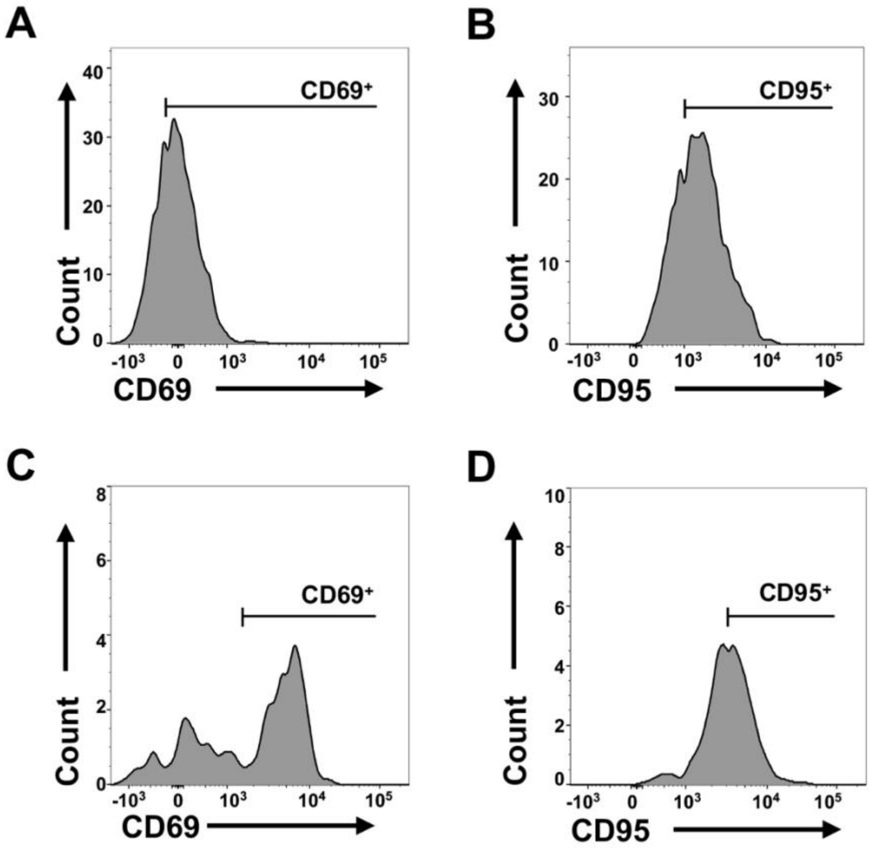

Figure S4. CD69 and CD95 expression in MAIT cells**.** Representative histograms of distribution of surface marker expression on $CD3^+$ $CD161^{hi}$ $V\alpha7.2^+$ MAIT cells in peripheral blood (A) and (B); and collagenase-digested liver tissues (C) and (D). Expression calculated from median fluorescent intensity (MFI) of gated regions.
